# Whole-Exome Sequencing Among School-Aged Children With High Myopia

**DOI:** 10.1001/jamanetworkopen.2023.45821

**Published:** 2023-12-01

**Authors:** Xiangyi Yu, Jian Yuan, Zhen Ji Chen, Kai Li, Yinghao Yao, Shilai Xing, Zhengbo Xue, Yue Zhang, Hui Peng, Gang An, Xiaoguang Yu, Jia Qu, Jianzhong Su

**Affiliations:** 1 National Engineering Research Center of Ophthalmology and Optometry, Eye Hospital, Wenzhou Medical University, Wenzhou, China; 2 National Clinical Research Center for Ocular Diseases, Eye Hospital, Wenzhou Medical University, Wenzhou, China; 3 Oujiang Laboratory, Zhejiang Laboratory for Regenerative Medicine, Vision and Brain Health, Wenzhou, Zhejiang, China; 4 Wenzhou Institute, University of Chinese Academy of Sciences, Wenzhou, China; 5 Institute of PSI Genomics, Wenzhou, China

## Abstract

**Importance:**

High myopia (HM) is one of the leading causes of visual impairment worldwide. Genetic factors are known to play an important role in the development of HM.

**Objective:**

To identify risk variants in a large HM cohort and to examine the implications of genetic testing of schoolchildren with HM.

**Design, Setting, and Participants:**

This cohort study retrospectively reviewed whole-exome sequencing (WES) results in 6215 schoolchildren with HM who underwent genetic testing between September 2019 and July 2020 in Wenzhou City, China. HM is defined as a spherical equivalent refraction (SER) of −6.00 diopters (D) or less. The study setting was a genetic testing laboratory and a multicenter school census. Data were analyzed from July 2021 to June 2022.

**Main Outcomes and Measures:**

The frequency and distribution of positive germline variants, the percentage of individuals with HM in both eyes, and subsequent variant yield for common high myopia (CHM; −8.00 D ≤ SER ≤ −6.00 D), ultra myopia (UM; −10.00 D ≤ SER < −8.00 D), and extreme myopia (EM; SER < −10.00 D).

**Results:**

Of the 6215 schoolchildren with HM, 3278 (52.74%) were male. Their mean (SD) age was 14.87 (2.02) years, including 355 students in primary school, 1970 in junior high school, and 3890 in senior high school. The mean (SD) SER was −7.51 (−1.36) D for the right eye and −7.46 (−1.34) D for the left eye. Among schoolchildren with HM, genetic testing yielded 271 potential pathogenic variants in 75 HM candidate genes in 964 diagnoses (15.52%). A total of 36 known variants were found in 490 HM participants (7.88%) and 235 protein-truncating variants (PTVs) in 506 participants (8.14%). Involved variant yield was significantly positively associated with SER (Cochran-Armitage test for trend Z = 2.5492; *P* = .01), which ranged from 7.66% in the CHM group, 8.70% in the UM group, to 11.90% in the EM group. We also found that primary school students with EM had the highest variant yield of PTVs (8 of 35 students [22.86%]), which was 1.77 and 4.78 times that of the UM and CHM, respectively.

**Conclusions and Relevance:**

In this cohort study of WES for HM, several potential pathogenic variants were identified in a substantial number of schoolchildren with HM. The high variation frequency in younger students with EM can provide clues for genetic screening and clinical examinations of HM to promote long-term follow-up assessment.

## Key Points

**Question:**

Is genetic testing necessary in the diagnosis of high myopia?

**Findings:**

This cohort study of whole-exome sequencing (WES) on 6215 schoolchildren with high myopia (HM) identified a total of 271 potential pathogenic variants in 75 HM candidate genes in 15.52% students. Subgroup analysis stratified by age and degree of refractive error revealed that the involved variants yield ranging from 7.66% in the common HM group, 8.70% in the ultra myopia group, 11.90% in the extreme myopia group to 22.86% in primary school students with extreme myopia.

**Meaning:**

This study revealed the genetic factor of high myopia, and is expected to guide future research and clinical diagnosis of HM.

## Teaser

This cohort study evaluates risk variants in a large high-myopia cohort and the implications of genetic testing of schoolchildren with high myopia.

## Introduction

Myopia, the most common cause of visual impairment, is a global public health concern due to its increasing prevalence.^1,2^ High myopia (HM) is generally defined as myopia with a spherical equivalent refractive (SER) error of less than or equal to −6.0 diopters (D). HM is a leading cause of impaired vision due to its association with increased risk of serious ocular complications, most notably retinal degeneration^3,4^ or even detachment.^5,6^ The prevalence of HM was reported to be 2% to 5% in American, Western European, and Australian populations^7^ compared with 4.5% to 38% in East Asian populations.^8,9,10,11^ A recent study predicted that visual impairment among preschool children will increase by 26% by 2060 with uncorrected refractive error comprising 69% of cases.^12^ Similar to many other common diseases, HM is a multifactorial disorder, involving interactions between genetic and environmental factors.^13,14^ Twin and family studies have demonstrated a high heritability for HM, which was estimated to be approximately 90%.^15,16^

In several early linkage studies and candidate gene studies for myopia, up to 50 loci and genes were identified before the genome-wide association studies (GWAS) era,^17,18,19,20,21,22,23,24^ but these could not be replicated in other related studies. In a GWAS performed using myopia as a dichotomous outcome or refractive error as a quantitative trait, a series of significant common genetic variants were identified in several loci,^25,26^ such as 11q24.1,^27^ 5p15,^28^ 4q25,^29^ 13q12,^30^ 21q22,^31^ 15q14,^32^ and 15q25.^33^ However, these loci account for only a limited fraction of the heritability for refractive error likely because of small effects not detected due to insufficient statistical power and causal variants, especially those of low allele frequency, not well captured by conventional GWAS. Compared with single-nucleotide variant (SNV) arrays, whole-exome sequencing (WES) offers additional opportunities to investigate rare coding variants that are not well tagged by array SNVs or imputation. Recently, WES has revealed variations in a few genes reported to be associated with HM, including *CCDC111*,^34^ *NDUFAF7*,^35^ *P4HA2*,^36,37^ *SCO2*,^38^ *UNC5D*,^39^ *BSG*,^40^ *ARR3*,^41,42^ *LOXL3*,^43,44^ *SLC39A5*,^45,46^ *LRPAP1*,^47^ *CTSH*,^47^ *CPSF1*,^48^ *TNFRSF21*,^49^ *DZIP1*, *XYLT1,*^50^ and *ZNF644*.^51,52^ On the other hand, HM has also been identified as a symptom of various forms of retinal dystrophies and systemic syndromes caused by several known genes, including *COL2A1*,^53^ *COL11A1*,^54^ *COL9A1*,^55^ and *COL9A2*^56^ responsible for Stickler syndrome (OMIM Nos. 108300, 604841, 614134, 614284) and *FBN1*^57^ for Marfan syndrome (OMIM No. 154700). Variants in these genes were detected in a few cases, with screening of additional HM cohorts expected to uncover more variants. WES studies in HM have made significant advancements by identifying new candidate genes for HM and highlighting the critical role of genetic factors in the development of this condition.^58^ For instance, a WES study involving 27 families has uncovered 201 candidate variants associated with HM.^59^ Additionally, our previous study has shown that population-based WES can discover functional risk alleles and provide important clues to elucidate the cause of HM.^60^ Therefore, large-scale genetic screening for school-aged children with HM could provide further insight into the intersecting contributions of biology and the environment.

In this study, we performed WES on 6215 school-aged children with HM from the Myopia Associated Genetics and Intervention Consortium (MAGIC) cohort to identify variants in known HM-associated genes and potentially contributory variants in HM genes. This study applied genetic testing to a larger population than previous reports. To our knowledge, this whole-exome germline genetic testing is the largest in a population with HM, highlighting the potential of genetic testing to improve diagnosis and management and to allow the definition of specific care pathways.

## Methods

### Study Design and Participants

The present study was approved by the ethics committee of the Wenzhou Medical University Affiliated Eye Hospital. Written informed consent conforming to the tenets of the Declaration of Helsinki and following the Guidance of Sample Collection of Human Genetic Diseases by the Ministry of Public Health of China was obtained from all participating individuals or their guardians before the study. The study adhered to the Strengthening the Reporting of Observational Studies in Epidemiology (STROBE) reporting guideline for cohort studies. A list of abbreviations used in the study is available in the eAppendix in Supplement 1.

As part of our ongoing efforts within MAGIC,^60^ a genetic analysis of refraction-confirmed myopia was conducted among schoolchildren in 10 districts in Wenzhou City, Zhejiang Province, China (eTable 1 in Supplement 2). MAGIC is a large-scale genomic consortium integrating myopia cohorts and sequencing data from different centers. Over the past several years, MAGIC has recruited approximately 10 000 Chinese schoolchildren with HM aged 6 to 18 years. For this cohort study, we developed a semiautomated vision examination and an information-entry pipeline, online eyesight status information management system,^61^ which involves manual inspection of automated refractometry data, automatic data import, and collaboration between clinicians and a statistician (eFigure 1 in Supplement 1). Certified technicians were trained at the Wenzhou Medical University Affiliated Eye Hospital with respect to standard procedures for determining visual acuity (VA) and autorefraction testing. Each school in the district was equipped with an autorefractometer (GoldEye RM-9000) and electronic logarithmic visual chart (GoldEye CM-1900C) by the Wenzhou Municipal Government to assess the prevalence of myopia. VA was evaluated using an E-type standard logarithmic visual chart at a distance of 5 m. Due to the large sample size, performing the criterion standard of cycloplegic refraction to diagnose myopia was not applicable. All students underwent noncycloplegic refraction testing using an automated refractometer, followed by complement subjective refraction testing for validation using an E-type standard logarithmic visual chart.^62^ Students who required corrective lenses were examined with and without eyeglasses, and naked eye refraction data were used to calculate the spherical equivalent.

### Outcomes

In this case-only cohort study, high myopia was defined as a VA of less than 1.0, and SER of both eyes was defined as sphere + (cylinder / 2) of −6.00 D or less. From September 2019 to July 2020, a total of 6215 school-agedchildren with HM in both eyes, including 355 primary school students (grades 1-6 in Chinese education system), 1970 junior high school students (grades 7-9), and 3890 senior high school students (grades 10-12) (eTable 1 in Supplement 2). Previous studies have reported that extreme myopia (EM) was associated with an increased risk of various ocular pathologic complications compared with common HM.^63^ Additionally, some substantial evidence suggested that EM is primarily associated with genetic factors rather than being solely caused by behavioral or environmental factors.^64^ Therefore, exploring the diagnostic utility across the different grades of HM among school ages is important for early diagnosis and efficient interventions in schoolchildren with HM. To this end, we further divided the participants into the 3 groups: common HM group (CHM, −8.00 D ≤ SER ≤ −6.00 D), ultra myopia group (UM, −10.00 D ≤ SER < −8.00 D), and EM group (SER < −10.00 D).^65^

### Whole-Exome Sequencing

A total of 6215 HM samples were sequenced on Illumina NovaSeq 6000 sequencers at Berry Genomics using the Twist Human Core Exome Kit. All samples were joint called together and were aligned to the consensus human genome sequence build GRCh37/hg19, and BAM files were processed using BWA^66^ and Sambamba 0.6.6.^67^ Genotype calling was performed using the Genome Analysis Toolkit’s (GATK) HaplotypeCaller.^68^

### Variant Interpretation

Variants were prefiltered so that only those passing the GATK VQSR (variant quality score recalibration) metric and those located outside of low-complexity regions remained. Low-certainty variant positions with a genotype depth (DP) less than 10 and genotype quality (GQ) less than 20 and heterozygous genotype calls with an allele balance of more than 0.8 or less than 0.2 were ignored. The annotation of variants was performed with Ensembl’s Variant Effect Predictor (VEP) version 99 for human genome assembly GRCh37.^69^ We used the VEP^70^ CADD, LOFTEE,^71^ and SpliceAI plugins to generate additional bioinformatic estimations of variant deleteriousness. Potentially pathogenic protein-truncating variants (PTVs) were classified as frameshift variant, splice acceptor variant, splice donor variant, stop gained, or start lost variants. Variant filtering included the following steps: (1) the present analysis focuses on rare variants with presumed large effect sizes. Therefore, we excluded variants that had minor allele frequencies (MAF) higher than 0.005 from 3 external exome sequence databases (1000 Genomes; National Heart, Lung, and Blood Institute Exome Sequencing Project; and gnomAD) and our MAGIC cohort. (2) We excluded variants not consistent with hereditary patterns, which were only 1-hit heterozygous variants in autosomal recessive (AR) genes and homozygous variants in autosomal dominant (AD) genes. Annotation of pathogenic (P), likely pathogenic (LP), variants of uncertain significance (VUS), benign (B), and likely benign (LB) for potentially pathogenic PTVs were performed by using ANNOtate VARiation software version 2020June,^72^ and pathogenicity was assigned according to 2015 American College of Medical Genetics (ACMG) criteria using InterVar,^73^ which is a computational implementation of expert panel recommendations for clinical interpretation of genetic variants (ACMG 2015 criteria).^74^

A total of 75 genes were included in this study (eTable 2 in Supplement 2), including 16 nonsyndromic HM genes, 27 genes of eye syndromes associated with HM, and 32 genes of systemic syndromes associated with HM (hereafter, we refer to this gene list as HM genes). All HM genes were extracted from OMIM,^75^ IMI-Myopia Genetics Report,^76^ and PubMed for articles published up to December 2022 using genetic relevant keywords (for example, gene, genetic, mutation, or variant) and “high myopia.” Variants from 75 genes were selected from the WES data set of the school-aged population with HM.

### Statistical Analysis

Involved variants yield was calculated as the percentage of patients with positive genetic test results among school-aged children with HM in each stratified group. Involved variants yield was also stratified by grade (primary, junior, and high school), SER (CHM, UM, and EM), and eyeballs (right and left). As a negative control compared with PTVs, variation rates for rare synonymous variants (MAF < 0.005 in in-house and external exome sequence databases) were computed among stratified groups. The significance of differences between each continuous groups (eg, CHM, UM, and EM) was assessed using the Cochran-Armitage test, and that of differences between categorical variables was assessed using the χ^2^ test. The threshold of multiple testing was set as *P* < .01 (.05 / 4 testings), which was calculated as the 5% type I error rate divided by the number of testings (right eye, left eye, PTV, and synonymous variants). R software version 3.6.1 (R Project for Statistical Computing) was used for analyses. Data were analyzed from July 2021 to June 2022.

## Results

### Cohort Information

Of the 6215 schoolchildren with HM, 3278 (52.74%) were male. Their mean (SD) age was 14.87 (2.02) years, and 355 participants (5.71%) were primary school students (aged ≤11 years); 1970 (31.70%) were junior school students (aged 12-14 years), and 3890 (62.59%) were high school students (aged ≥15 years). The mean (SD) SER was −7.51 (−1.36) D for the right eye and −7.46 (−1.34) D for the left eye. Detailed demographic information is presented in eTable 1 in Supplement 2.

### Variational Spectrums Revealed for Known HM-Related Genes

**Figure 1.**
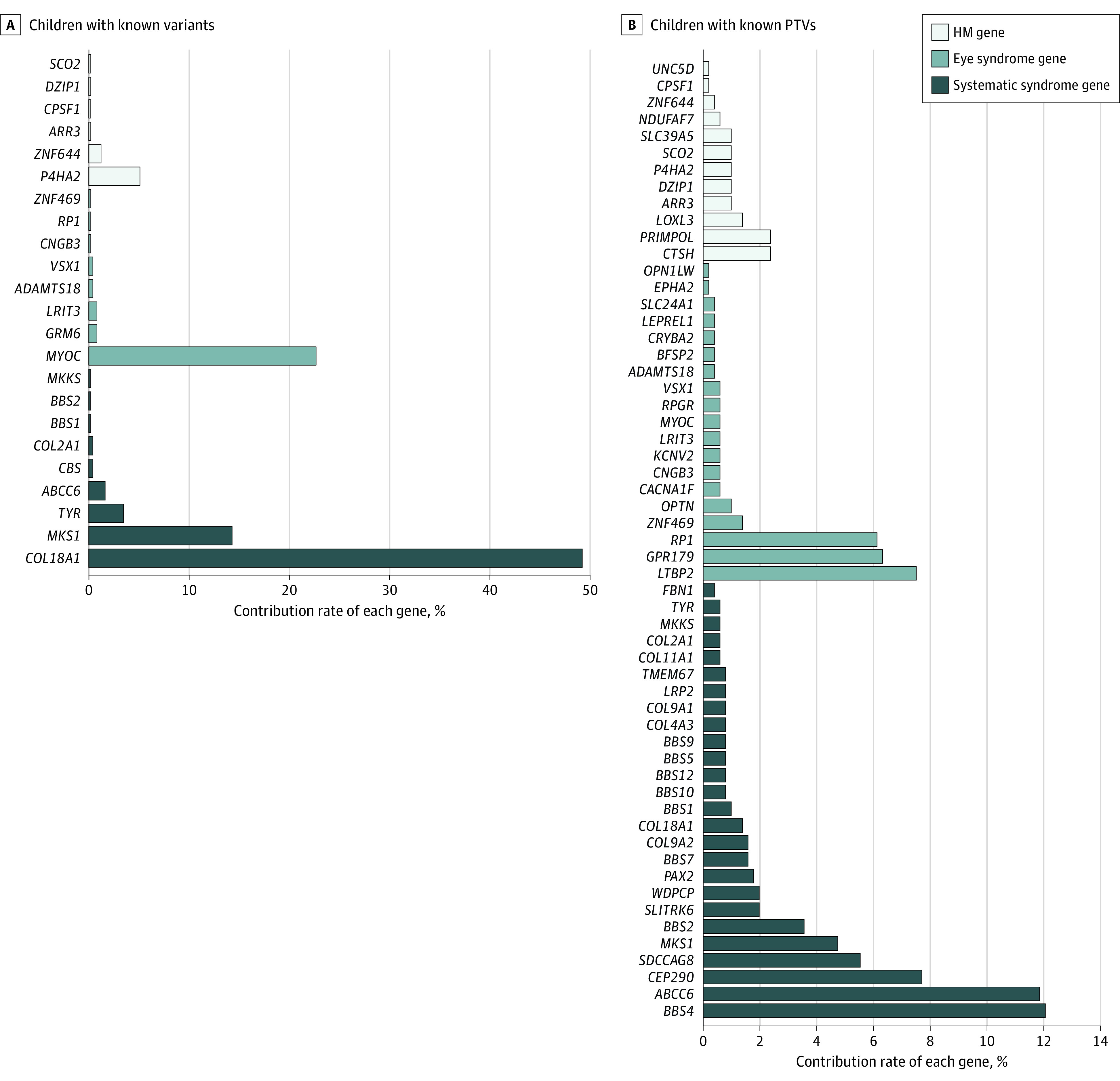
Distribution of Varied Genes in the 409 Schoolchildren With Known Variants and 506 Schoolchildren With Protein-Truncating Variants (PTV) Who Received a Probable Molecular Diagnosis After Whole-Exome Sequencing Genetic Testing Genes without known variants and rare PTVs are not shown. HM indicates high myopia.

Through the analysis of the exome sequencing data of 6215 individuals with HM, a total of 271 potential pathogenic variants in 59 of 75 candidate genes were identified in 964 (15.52%) schoolchildren with HM, including 36 known variants in 490 participants (7.88%) and 235 newly identified PTVs in 506 participants. Of 271 variants, 237 P/LP variants, 32 VUS, and 2 B variants were identified using ACMG guidelines (eTable 3 in Supplement 2). Among 36 previously reported variants in 490 participants, 9 variants in 6 HM genes were identified in 34 participants (0.55%), 10 variants in 8 eye syndrome genes were detected in 125 participants (2.01%), and 17 variants in 9 systemic syndrome genes were found in 337 participants (5.42%) (eTable 4, eTable 5, and eTable 6 in Supplement 2). The most frequently varied genes were *COL18A1* (c.4318G>A and c.3523_3524del), accounting for 49.18% of the diagnoses, respectively (241 of 490 participants) (Figure 1A).

**Figure 2.**
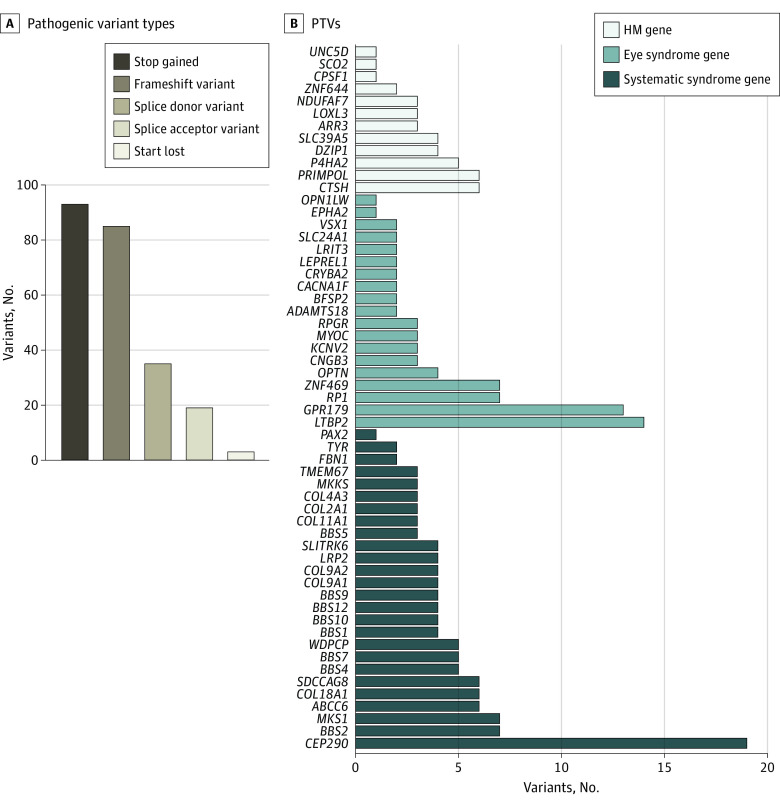
Rare Pathogenic Variants Identified in High Myopia (HM) Candidate Genes A, Pathogenic variant types in HM candidate genes detected in schoolchildren with HM. B, Number of protein-truncating variants (PTV) identified in HM candidate genes. Bar plot shows the frequency of PTV in nonsydromic HM genes, eye syndrome genes and systemic syndrome genes. Genes without rare PTVs are not shown.

A total of 235 potentially P PTVs were identified in 506 of 6215 individuals (8.14%) with HM. Among them, 39 PTVs in HM genes were identified in 63 of 6215 participants (1.01%), 75 PTVs of eye syndrome genes in 132 of 6215 participants (2.12%) and 121 PTVs of systemic syndrome genes in 327 of 6215 participants (5.26%) (eTable 7, eTable 8, and eTable 9 in Supplement 2). Of the 57 genes with PTVs, variants in *BBS4* were the most common, accounting for 12.06% of individuals (61 of 506 participants) with PTVs (Figure 1B). Of the 235 potentially P variants, 171 were only found once (ie, in a single student), whereas 64 occurred in 2 or more unrelated students, suggesting substantial genetic heterogeneity in HM loci. Variant types included frameshift (85 of 235 participants), splice acceptor (19 of 235 participants), splice donor (35 of 235 participants), stop gained (93 of 235 participants) and start lost (3 of 235 participants) variants (Figure 2A). We identified 19 PTVs within *CEP290* and 14 PTVs in *LTBP2* (Figure 2B). These P pathogenic PTVs in HM-related genes have not been observed in public exome sequence databases. They included 14 heterozygous variants in 6 genes (*COL11A1, COL2A1, FBN1, LRP2, UNC5D*, and *ZNF644*) and 2 homozygous variants in *OPN1LW* and *RPGR* genes (Table). The most common variant in our study was a missense variant (NC_000021:c.4318G>A; p.Asp1440Asn) in *COL18A1*, which was detected in 3.86% (240 of 6215 participants) of our cohort.

**Table.** Overview of PTVs in HM Candidate Genes

Variant	Gene	Consequences	Transcript	cDNA change	Residue change	LOFTEE	MAF	Status	Sample ID
chr1_91405539	ZNF644	Stop gained	ENST00000370440	c.1372C>T	p.Arg458Ter	HC	8.05 × 10^−05^	HET	19BY19988
chr1_91447871	ZNF644	Frameshift variant	ENST00000370440	c.38_39del	p.Lys13IlefsTer2	HC	8.05 × 10^−05^	HET	19BY16965
chr1_103449692	COL11A1	Splice donor variant	ENST00000370096	c.2208 + 2T>C	NA	HC	8.05 × 10^−05^	HET	19BY08582
chr1_103491076	COL11A1	Splice donor variant	ENST00000370096	c.897 + 696G>A	NA	HC	8.05 × 10^−05^	HET	19BY06615
chr2_170002290	LRP2	Stop gained	ENST00000263816	c.12955C>T	p.Arg4319Ter	HC	8.05 × 10^−05^	HET	19BY04713
chr2_170034316	LRP2	Frameshift variant	ENST00000263816	c.10389dup	p.Ile3464HisfsTer33	HC	8.05 × 10^−05^	HET	19BY04553
chr2_170044720	LRP2	Stop gained	ENST00000263816	c.9088C>T	p.Gln3030Ter	HC	8.05 × 10^−05^	HET	19BY07153
chr2_170063503	LRP2	Stop gained	ENST00000263816	c.6727C>T	p.Arg2243Ter	HC	8.05 × 10^−05^	HET	19BY11969
chr8_35093311	UNC5D	Frameshift variant	ENST00000404895	c.1426G>T	p.Glu476Ter	HC	8.05 × 10^−05^	HET	19BY02740
chr12_48370913	COL2A1	Stop gained	ENST00000380518	c.3299C>G	p.Ser1100Ter	HC	8.05 × 10^−05^	HET	19BY04616
chr12_48380199	COL2A1	Stop gained	ENST00000380518	c.1447G>T	p.Gly483Ter	HC	8.05 × 10^−05^	HET	19BY06672
chr12_48383582	COL2A1	Stop gained	ENST00000380518	c.1030C>T	p.Arg344Ter	HC	8.05 × 10^−05^	HET	19BY04804
chr15_48829865	FBN1	Stop gained	ENST00000316623	c.679C>T	p.Gln227Ter	HC	8.05 × 10^−05^	HET	19BY12499
chr15_48829882	FBN1	Frameshift variant	ENST00000316623	c.661del	p.Cys221ValfsTer109	HC	8.05 × 10^−05^	HET	19BY06899
chrX_153416181	OPN1LW	Frameshift variant	ENST00000369951	c.168del	p.Ser57ValfsTer4	HC	1.61 × 10^−04^	HOM	19BY20410
chrX_38176655	RPGR	Frameshift variant	ENST00000378505	c.532del	p.Ser178ValfsTer9	HC	1.61 × 10^−04^	HOM	19BY06620

Abbreviations: cDNA, complementary DNA; HC, high confidence; HET, heterozygous; HOM, homozygous; MAF, minor allele frequency; NA, not applicable.

### Variant Yield by Severity of Myopia

**Figure 3.**
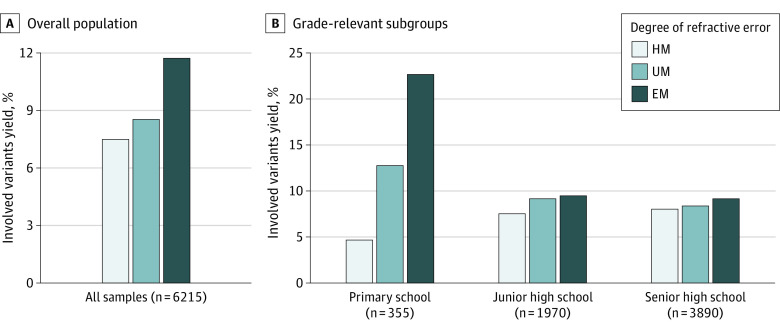
Proportion of Schoolchildren With High Myopia (HM) Carrying Protein-Truncating Variants in the Overall Study Population and Grade Relevant Subgroups Spherical refraction ranges are as follows: high myopia, −8 D to −6 D; ultra myopia (UM), −10 D to −8 D; extreme myopia (EM), less than −10 D. Participants were divided into 3 subgroups according to grade when they received genetic testing.

Next, we quantified the involved variants yield (defined as the percentage of cases) to HM attributable to PTVs in well-characterized HM genes. The analysis revealed that the proportion of participants with PTVs varied across HM conditions and grade groups. The involved variants yield showed a positive association with SER, ranging from 7.66% in the CHM group, 8.70% in the UM group to 11.90% in the EM group, with a statistically significant increasing trend (Cochran-Armitage test for trend Z = 2.5492; *P* = .01, Figure 3A). The trend was also seen in the independent cohorts of people with HM in either the left or the right eye alone (eFigure 2 in Supplement 3). Notably, high SER was significantly associated with a higher involved variants yield in the primary students (Cochran-Armitage test for trend Z = 3.8848; *P* < .001) but not in other grades (Cochran-Armitage test for trend Z = 1.299; *P* = .19 for junior high school, and Cochran-Armitage test for trend Z = 1.0135; *P* = .31 for senior high school) (Figure 3B). The primary students referred for EM had the highest yield (8 of 35 individuals [22.86%]), which was 1.77 and 4.78 times higher than the UM (12.90%; χ^2^_1_ = 0.52803; *P* = .16) and CHM (4.78%; χ^2^_1_ = 12.318; *P* < .001), respectively. This trend was consistent across groups divided by the SER of either eye (eFigure 3 in Supplement 3). However, as the negative control, the trend was not statistically significant for synonymous variants (eFigure 4 in Supplement 3).

## Discussion

In this study, we tested the historic utility of WES in 6215 school-aged students with HM. The association of HM with ocular and systemic diseases has been investigated in several previous studies. For example, Marr et al^77^ found that 56% of children with HM had simple symptoms of HM, 25% had ocular abnormalities, and 19% had systemic disorders. Another study found variants in genes known to be involved in retinal disease in about a quarter of the participants with early-onset HM.^78,79^ Therefore, 75 genes were included in the present study, including 16 nonsyndromic HM genes, 27 genes of eye syndromes, and 32 genes of systemic syndromes associated with HM, such as Stickler syndrome, Marfan syndrome, and Knobloch syndrome.

Systematic analysis of variants in the 75 candidate genes showed that genetic testing had a total involved variants yield of 15.52%, including 36 known variants in 490 participants and 235 PTVs in 506 participants. The discovery of variants previously obtained in other studies demonstrated the efficiency of a genetic approach to find pathogenic variants in our cohort. In our cohort, 36 known variants were found in a total of 490 patients (7.88%), including a stop-gain variant (NM_004312:c.298C>T; p.Arg100Ter) in *ARR3*^41^ found in 1 patient. *ARR3* is associated with early-onset HM in a unique X-linked female-limited inheritance.^80^ The most common variant in our study was a missense variant (NC_000021:c.4318G>A; p.Asp1440Asn) in *COL18A1*, which was detected in 3.86% (240 of 6215 participants) of our cohort. Variants in *COL18A1* have been identified in patients with Knobloch syndrome, an inherited disorder characterized by HM, retinal detachment, and occipital defects.^81,82^

PTVs result in reduced or abolished protein function and are thus considered to be the most deleterious variants.^83^ PTVs in HM candidate genes were detected in 506 patients (8.14%), including *ZNF644*. This is a zinc finger transcription factor expressed in the retina and RPE, which has been suggested to play a role in the development of HM because variants in *ZNF644* have been found in patients with HM.^51,52^ In the present study, we found 2 PTVs in *ZNF644* (c.1372C>T and c.38_39del), which have not been recorded in the gnomAD.^84^ We also found 3 previously unreported stop-gained variants (c.3299C>G, c.1447G>T, and c.1030C>T) and 3 splice-donor variants (c.2208 + 2T>C, c.898-972G>A, and c.897 + 696G>A) in *COL11A1*. Stickler syndrome is caused by variants of 2 genes,^85^ and is clinically characterized by HM, which is the most common cause of inherited retinal detachment.^86^ These results suggested that WES could find potential pathogenic variants and provide new genetic evidence for HM candidate genes in our study.

With the increase in the number of high-throughput sequencing projects generating vast amounts of data and the innovation of more statistical methods, the occurrence and development of HM can be systematically explored. On the one hand, our research showed the potential of large-scale WES in identifying new variants of high myopia. On the other hand, a detailed spherical refraction and grade (age)–stratified study implied the pattern of efficiency of genetic testing in schoolchildren with HM. In our study, the detection rate of PTVs increased with the severity of myopia, specific to the primary school group. Although the diagnostic efficacy of WES in the prevention of HM needs to be further evaluated, more severe myopia presentation with more positive genetic test results was found in younger children, suggesting the importance of genetic testing for early diagnosis and prevention of myopia. Early identification of positive variants may encourage more frequent assessment of high myopia progression and screening of at-risk family members, which can improve behavioral intervention and clinical management for schoolchildren with HM. Taken together, these findings emphasize the potential value of genetic testing in resolving clinical diagnostic challenges in HM.

### Limitations

Although this study was based on grade (age) and spherical equivalent, detailed ocular and systemic examinations, such as eye axis and fundus photography, were not performed. Therefore, additional ocular biological factors should be considered in future research, which could further provide genetic diagnoses across diverse clinical categories, enabling the identification of novel phenotypic extensions. Furthermore, this study lacks follow-up data over several years, and there is a possibility that HM may progress into ultra or extreme myopia in some patients. A clearer conclusion would be reached if the genetic results could be combined with detailed clinical examinations, especially long-term follow-up assessment for schoolchildren with specific variants. Noncycloplegic assessment of refractive error in children overestimates myopia. Cycloplegic autorefraction could be implemented as an efficient approach in the follow-up study. To ensure data reliability, future multiyear follow-up studies are warranted. In addition, WES currently has limited ability to detect genomic imbalances and does not assess variants in noncoding regions of the genome, leaving additional blind spots. Physician knowledge of these technical limitations is important as WES is increasingly being incorporated into clinical practice.

## Conclusions

In summary, WES screening can reveal important HM candidate genes at once and allows periodic reevaluation of the sequence data to identify new disease genes. In this study, WES also showed potential diagnostic value for different categories of schoolchildren with HM, yielding the highest contribution rate of pathogenic variants in primary school students. Our study illustrated the ability of genetic testing to identify potentially pathogenic variants in HM and reveal new genes contributing to myopia.

## Notes

Supplement 1. **eFigure 1.** Workflow Chart and Study Profile **eFigure 2.** Proportion of HM Cases Carrying Rare PTVs in Overall Study Population **eFigure 3.** Proportion of HM Cases Carrying Rare PTVs in Primary School, Junior School, and High School **eFigure 4.** Proportion of HM Cases Carrying Rare Synonymous Variants in Primary School, Junior School, and High School

Supplement 2. **eTable 1.** Catchment Areas and Schoolchildren Included in the Study **eTable 2.** Genes Related to HM According to the OMIM **eTable 3.** ACMG Classification of 271 Potential Pathogenic Variants **eTable 4.** Demographic and Genetic Characteristics of HM Individual With Known Variations in HM Genes **eTable 5.** Demographic and Genetic Characteristics of HM Individual With Known Variations in Eye Syndrome Genes **eTable 6.** Demographic and Genetic Characteristics of HM Individual With Known Variations in Systemic Syndrome Genes **eTable 7.** All Potential Pathogenic PTVs in HM Genes **eTable 8.** All Potential Pathogenic PTVs in Eye Syndrome Genes **eTable 9.** All Potential Pathogenic PTVs in Systemic Syndrome Genes

Supplement 3.
Nonauthor Collaborators

Supplement 4.
Data Sharing Statement
