# Supplementary material for: Whole-Exome Sequencing Among School-Aged Children With High Myopia
Source: JAMA Netw Open. 2023 Dec 1;6(12):e2345821. doi: 10.1001/jamanetworkopen.2023.45821 (PMC10692858; doi:10.1001/jamanetworkopen.2023.45821)
Supplement: Supplement 1. — eFigure 1. Workflow Chart and Study Profile eFigure 2. Proportion of HM Cases Carrying Rare PTVs in Overall Study Population eFigure 3. Proportion of HM Cases Carrying Rare PTVs in Primary School, Junior School, and High School eFigure 4. Proportion of HM Cases Carrying Rare Synonymous Variants in Primary School, Junior School, and High School [file jamanetwopen-e2345821-s001.pdf]

Supplemental Online Content

Yu X, Yuan J, Chen Z, et al; Myopia Associated Genetics and Intervention Consortiums. Whole-exome sequencing among school-aged children with high myopia. *JAMA Netw Open*. 2023;6(12):e2345821. doi:10.1001/jamanetworkopen.2023.45821

eFigure 1. Workflow Chart and Study Profile
eFigure 2. Proportion of HM Cases Carrying Rare PTVs in Overall Study Population
eFigure 3. Proportion of HM Cases Carrying Rare PTVs in Primary School, Junior School, and High School
eFigure 4. Proportion of HM Cases Carrying Rare Synonymous Variants in Primary School, Junior School, and High School

This supplemental material has been provided by the authors to give readers additional information about their work.

© 2023 Yu X et al. *JAMA Network Open.*

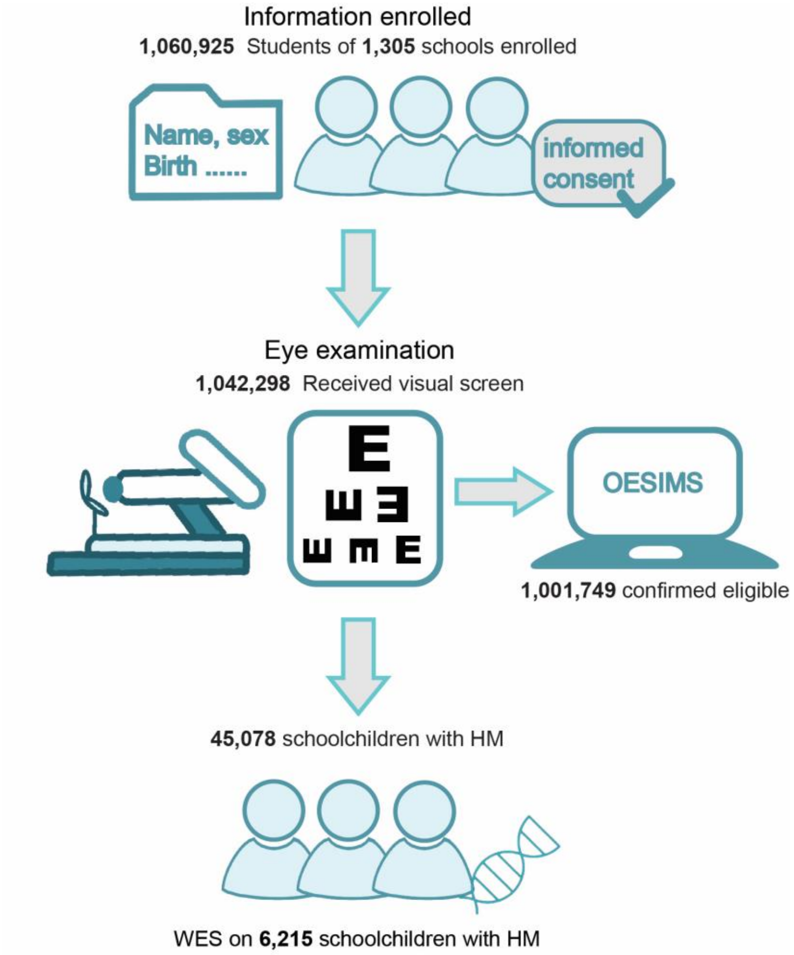

eFigure 1. Workflow Chart and Study Profile

© 2023 Yu X et al. *JAMA Network Open.*

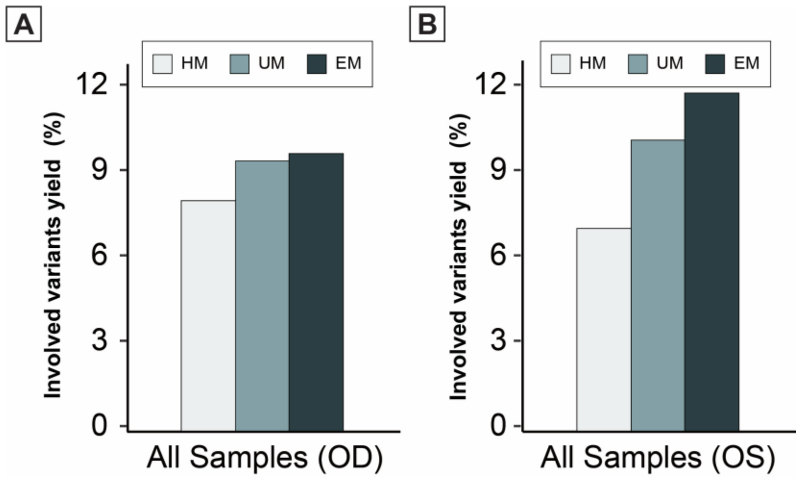

eFigure 2. Proportion of HM Cases Carrying

Rare PTVs in Overall Study Population. (A) OD: right eyes. (B) OS: left eyes.

© 2023 Yu X et al. *JAMA Network Open.*

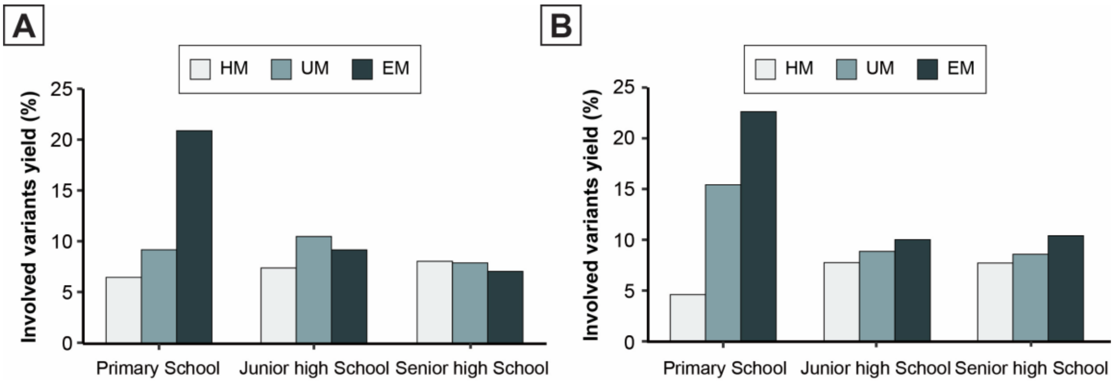

eFigure 3. Proportion of HM Cases Carrying Rare PTVs in Primary School, Junior School, and High School. The participants were divided into three subgroups according to the grade when they received genetic test. (A) OD: right eyes. (B) OS: left eyes.

© 2023 Yu X et al. *JAMA Network Open.*

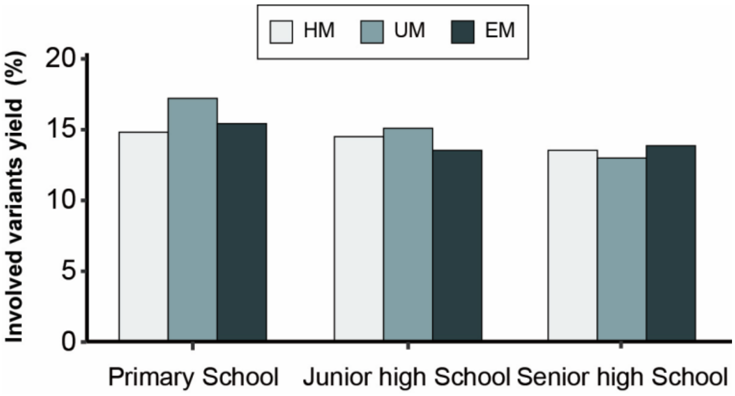

eFigure 4. Proportion of HM Cases Carrying Rare Synonymous Variants in Primary School, Junior School, and High School. The participants were divided into three subgroups according to the grade when they received genetic test.

© 2023 Yu X et al. *JAMA Network Open.*
