# Supplementary material for: Whole-Exome Sequencing Among School-Aged Children With High Myopia
Source: JAMA Netw Open. 2023 Dec 1;6(12):e2345821. doi: 10.1001/jamanetworkopen.2023.45821 (PMC10692858; doi:10.1001/jamanetworkopen.2023.45821)
Supplement: Supplement 3. — Nonauthor Collaborators [file jamanetwopen-e2345821-s003.pdf]

Supplemental Online Content: Nonauthor Collaborators
*First name, last name, and suffix (if applicable) are required and will appear in PubMed.

*Group Name(s): Myopia Associated Genetics and Intervention Consortium							
***First Name and Middle Initial(s)**	***Last Name**	***Suffix (eg, Jr, III)**	Academic Degrees	Institution	Location (city, state/province, country)	Role or Contribution, eg, chair, principal investigator	Group (if more than 1 Group listed in the byline) and/or Subgroup (eg, Steering Committee)
Liangde	Xu		PhD	Wenzhou Medical University	WenZhou	principal investigator	
Hong	Wang		PhD	Wenzhou Medical University	WenZhou	principal investigator	
Meng	Zhou		PhD	Wenzhou Medical University	WenZhou	principal investigator	
Jie	Sun		PhD	Wenzhou Medical University	WenZhou	principal investigator	
Hao	Chen		MD	Eye Hospital, Wenzhou Medical Universit	WenZhou	principal investigator	
Fan	Lyu		MD	Eye Hospital, Wenzhou Medical Universit	WenZhou	principal investigator	
Xinting	Liu		MD	Eye Hospital, Wenzhou Medical Universit	WenZhou	principal investigator	
Jinhua	Bao		MD	Eye Hospital, Wenzhou Medical Universit	WenZhou	principal investigator	
Nan	Wu		MD	Peking Union Medical College Hospital	Beijing	principal investigator	
