# Supplementary material for: Whole-Exome Sequencing Among School-Aged Children With High Myopia
Source: JAMA Netw Open. 2023 Dec 1;6(12):e2345821. doi: 10.1001/jamanetworkopen.2023.45821 (PMC10692858; doi:10.1001/jamanetworkopen.2023.45821)
Supplement: Supplement 4. — Data Sharing Statement [file jamanetwopen-e2345821-s004.pdf]

Data Sharing Statement

Yu. Whole-Exome Sequencing Among School-Aged Children With High Myopia. *JAMA Netw Open*. Published December 01, 2023. doi:10.1001/jamanetworkopen.2023.45821

Data
Data available: No

Additional Information
Explanation for why data not available: Individual-level data are not publicly available due to ethical and legal restrictions related to the Wenzhou Medical University. The datasets of genotype information are available from the corresponding author on request.
